# Protective Effects of Exogenous Melatonin Administration on White Fat Metabolism Disruption Induced by Aging and a High-Fat Diet in Mice

**DOI:** 10.3390/antiox13121500

**Published:** 2024-12-09

**Authors:** Dongying Lv, Yujie Ren, Jiayan Chen, Ziyao Pang, Yaxuan Tang, Lizong Zhang, Laiqing Yan, Xiufeng Ai, Xiaoping Xv, Dejun Wang, Zhaowei Cai

**Affiliations:** 1 Laboratory Animal Research Center, Academy of Chinese Medical Sciences, Zhejiang Chinese Medical University, Hangzhou 310053, China; 20211039@zcmu.edu.cn (D.L.); arianatang5113@163.com (Y.T.); ryan_zlz@163.com (L.Z.); 20191015@zcmu.edu.cn (X.A.); xxp@zcmu.edu.cn (X.X.); 2 School of Pharmacy, Zhejiang Chinese Medical University, Hangzhou 310053, China; ruiqing1109@126.com (Y.R.); 18407878185@163.com (J.C.); pang3056527363@163.com (Z.P.); 3 State Key Laboratory of Animal Biotech Breeding, Key Laboratory of Animal Genetics, Breeding and Reproduction of the Ministry of Agriculture, College of Animal Science and Technology, China Agricultural University, Beijing 100193, China; laiqingyan@cau.edu.cn; 4 Zhejiang Key Laboratory of Blood-Stasis-Toxin Syndrome, Zhejiang Chinese Medical University, Hangzhou 310053, China

**Keywords:** obesity, aging, eWAT, lipidomics, melatonin, inflammation

## Abstract

Obesity has emerged as a major risk factor for human health, exacerbated by aging and changes in dietary habits. It represents a significant health challenge, particularly for older people. While numerous studies have examined the effects of obesity and aging on fat metabolism independently, research on their combined effects is limited. In the present study, the protective action against white fat accumulation after a high-fat diet (HFD) exerted by exogenous melatonin, a circadian hormone endowed with antioxidant properties also involved in fat metabolism, was investigated in a mouse model. For this purpose, a battery of tests was applied before and after the dietary and melatonin treatments of the animals, including epididymal white adipose tissue (eWAT) histological evaluations, transcriptomic and lipidomic analyses, real-time PCR tests, immunofluorescence staining, Western blot, the appraisal of serum melatonin levels, and transmission electron microscopy. This study found that aged mice on a high-fat diet (HFD) showed increased lipid deposition, inflammation, and reduced antioxidant glutathione (GSH) levels compared to younger mice. Lipidomic and transcriptomic analyses revealed elevated triglycerides, diglycerides, ceramides, and cholesterol, along with decreased sphingomyelin and fatty acids in eWAT. The genes linked to inflammation, NF-κB signaling, autophagy, and lipid metabolism, particularly the melatonin and glutathione pathways, were significantly altered. The aged HFD mice also exhibited reduced melatonin levels in serum and eWAT. Melatonin supplementation reduced lipid deposition, increased melatonin and GSH levels, and upregulated AANAT and MTNR1A expression in eWAT, suggesting that melatonin alleviates eWAT damage via the MTNR1A pathway. It also suppressed inflammatory markers (e.g., TNF-α, NLRP3, NF-κB, IL-1β, and CEBPB) and preserved mitochondrial function through enhanced mitophagy. This study highlights how aging and HFD affect lipid metabolism and gene expression, offering potential intervention strategies. These findings provide important insights into the mechanisms of fat deposition associated with aging and a high-fat diet, suggesting potential intervention strategies.

## 1. Introduction

With the significant increase in human life expectancy, the world’s population is aging rapidly [1]. Adipose tissue, the primary site of energy storage, is crucial for maintaining energy homeostasis and metabolic health, processes often disrupted by aging. Notably, adipose tissue is one of the few organs where targeted interventions can impact both lifespan and health span [2]. Recent studies have highlighted the vulnerability of adipose tissue to aging. Age-associated immune responses are initially observed in the white adipose tissue (WAT) of both mice and humans [3]. The most significant age-related transcriptomic changes in mice occur in the epididymal WAT (eWAT) [4]. It is now understood that WAT plays a pivotal role in regulating cardiovascular disease, energy storage, and systemic metabolic homeostasis [5]. Emerging evidence suggests that local inflammation in adipose tissue is a major contributor to systemic inflammation [6]. A recent study found that the eWAT exhibits the strongest inflammatory levels compared to inguinal WAT and brown adipose tissue in 18-month-old mice [7]. Oxidative stress, a key driver of adipose tissue aging, leads to a decline in adipocyte function, exacerbated local inflammation, and metabolic dysfunction [8]. Reactive oxygen species (ROS) generated by oxidative stress damage adipocytes and activate pro-inflammatory signaling pathways, accelerating cellular aging and tissue damage [9]. Consequently, these changes can negatively impact both adipose tissue and the overall metabolic state and immune response by secreting various inflammatory factors and hormones [10]. Therefore, studying the healthy metabolism of eWAT during aging is significant.

Among poor dietary habits, a high-fat diet (HFD) is a major contributor to obesity and metabolic disorders [11]. Studies have shown that HFD significantly alters lipid composition and gene expression profiles in adipose tissue, leading to increased lipid species such as triglycerides and ceramides, and altered gene expression related to inflammation, autophagy, and lipid metabolism [12]. Adipose tissue is classified into two types: brown adipose tissue (BAT) and WAT [13]. The morphological differences between these types result in distinct functions: BAT primarily regulates body temperature through non-shivering thermogenesis, whereas WAT and eWAT play a crucial role in obesity-related metabolic disorders [14]. WAT is the main site of inflammation in obesity and contains numerous immune cells, including macrophages, eosinophils, and natural killer (NK) cells, which are involved in the regulation of immunity and thermogenesis. A high-fat diet induces oxidative stress in adipose tissue, which may impair adipose tissue metabolism by promoting triglyceride accumulation [15]. Excess triglyceridestorage in adipose tissue and adipocyte–macrophage interactions initiate a cycle of macrophage recruitment and the NF-κB-dependent expression of pro-inflammatory cytokines such as interleukin-1β (IL-1β), interleukin-6 (IL-6), and tumor necrosis factor-α (TNF-α) [16]. In addition to being a primary site of inflammation, WAT is also the major energy storage site in mammals. The mitochondria, the primary sites of cellular energy production, are major targets of oxidative stress, as they are the centers of cellular redox reactions. Mitochondrial oxidative stress is elevated during aging, leading to mitochondrial dysfunction [17]. Mitochondrial dysfunction is closely associated with cellular inflammatory responses. As mitochondrial activity declines with age, the accumulation of damaged mitochondria increases over time [18]. Mitophagy, the selective removal of damaged mitochondria, is crucial for maintaining the health of adipose tissue. Impaired mitophagy can exacerbate inflammatory responses, promote adipocyte hypertrophy, and have significant implications for obesity and energy metabolism [19]. Age is a significant risk factor for obesity, and aging individuals may be more susceptible to the adverse effects of an HFD due to impaired metabolic function and reduced antioxidant capacity [20,21]. Therefore, protecting the metabolic health of eWAT in aging individuals is of significant interest.

The chemical name of melatonin is N-acetyl-5-methoxytryptamine, a molecule attracting interest in these kinds of investigations, which is present in almost all organisms. While it is commonly associated with the pineal gland, melatonin is also widely distributed across mammalian tissues and organs, particularly within the mitochondria [22,23,24]. The synthesis of melatonin involves several steps: first, tryptophan is converted into 5-hydroxytryptophan by tryptophan hydroxylase (TPH), followed by its conversion into 5-hydroxytryptamine (5-HT) via aromatic amino acid decarboxylase. Subsequently, serotonin N-acetyltransferase (SNAT/AANAT) transforms serotonin into N-acetyl-5-hydroxytryptophan, which is then converted into melatonin by hydroxyindole-O-methyltransferase (HIOMT/AANAT) [25]. Research shows that serum melatonin levels decline with age, suggesting that melatonin plays a significant role in the aging process [26]. Additionally, many studies have demonstrated that melatonin can protect animals from age-related metabolic impairment. Early research found that exogenous melatonin inhibits brain and nerve aging in mice [27,28]. Recent findings suggest that exogenous melatonin also protects other tissues, such as maintaining the integrity of vascular endothelium in aging mice through intraperitoneal injection [29]. Melatonin was initially identified as a regulator of biological rhythms. However, it is now known to perform antioxidant and anti-inflammatory functions by modulating the expression of antioxidant enzymes and inflammation-related genes [30]. Studies indicate that melatonin plays a role in lipid metabolism and inflammation suppression, particularly by downregulating inflammatory factors in the NF-κB signaling pathway to mitigate inflammatory responses. It also enhances antioxidant capacity by elevating glutathione (GSH) levels and upregulating enzymes like glutathione peroxidase (GPX) [31]. Moreover, exogenous melatonin supplementation has been shown to significantly reduce body weight [32] and lower triglyceride levels in obese rats and mice. It also protects the hippocampus and bone tissue in obese mice and reduces inflammation in adipose tissue [33]. Based on these findings, we hypothesize that melatonin is involved in the protection of eWAT and contributes to the anti-inflammatory processes in aged mice.

While several studies have investigated the effects of aging on adipose tissue, the synergistic effects of aging and a high-fat diet on epididymal adipocyte metabolism remain underexplored. This study aimed to investigate how aging and a high-fat diet jointly affect epididymal adipocyte metabolism in mice to elucidate their synergistic mechanisms and to assess the potential protective effects of melatonin. By systematically analyzing changes in lipid metabolism, inflammatory responses, and cell function in the epididymal adipose tissue of aged mice exposed to an HFD, we aim to elucidate how these factors collectively contribute to fat metabolism disorders and to explore the regulatory role of melatonin, which we have previously investigated in alleviating oxidative stress and associated metabolic disorders.

## 2. Materials and Methods

### 2.1. Ethics Statement

All animal studies were conducted in accordance with the guidelines established by the Animal Care and Use Committee protocols and were approved by the Ethics Committee of the Zhejiang Chinese Medical University of China (Approval No. IACUC-20230918-02).

### 2.2. Mice and Treatments

We conducted two batches of experiments using male C57BL/6J mice. Young (2-month-old) mice were purchased from Qi Zhen Animal Technology Co., Ltd. (Hangzhou, China), and aged (18-month-old) mice were obtained from Hua Chuang Xin Nuo Medicine Technology Co., Ltd. (Taizhou, China). All the mice were housed under specific pathogen-free conditions, with a 12 h light–dark cycle, a temperature of 22 ± 5 °C, and ad libitum access to food and water. The animals were housed in groups of 3–4 per cage. They had the same amount of freedom of movement in accordance with the National Standard—Laboratory Animals—General Requirements for Animal Experiments. In this way, all the animals, young and old, subjected to HFD and treated with melatonin, lived under the same conditions in terms of motor activity. In the first experiment, 28 age-matched male mice were randomly assigned to four groups (n = 7 per group): (1) young control (YC), (2) young high-fat diet (YH), (3) old control (OC), and (4) old high-fat diet (OH). Groups 1 and 2 consisted of young mice, while groups 3 and 4 comprised aged mice. Groups 1 and 3 were fed a normal chow diet, whereas groups 2 and 4 were fed a high-fat diet (21% fat, 0.21% cholesterol; D12079B, Research Diets Inc., New Brunswick, NJ, USA). In the second experiment, 21 aged male mice were randomly assigned to three groups (n = 7 per group): (5) old control (OC), (6) old high-fat diet (OH), and (7) old high-fat diet + melatonin (OHm). All the mice in this experiment were 17–18 months old. The mice in group 6 were fed a high-fat diet, and the mice in group 7 received 0.4 mg/mL melatonin solution (HY-B0075, MCE) in addition to the high-fat diet, as referenced in the published literature [34,35]. Water bottles containing the melatonin solution were wrapped in light-tight aluminum foil and changed daily. Body weights and food intake were recorded weekly. After 16 weeks of feeding, the mice were euthanized under sodium pentobarbital anesthesia. Blood samples were collected, and the eWAT was excised and weighed. The eWAT was then immediately frozen in liquid nitrogen and stored at −80 °C for subsequent analysis.

### 2.3. Histological Analysis

The eWAT of the mice was fixed in 10% paraformaldehyde, rinsed with running water, dehydrated, and embedded for sectioning. Sections of approximately 6 μm thickness were obtained, flattened in warm water at 45 °C, mounted on slides, dried at 60 °C, deparaffinized, and stained with hematoxylin and eosin (H&E) using an automated slide stainer. The slides were mounted with neutral resin. A digital pathology slide scanning system was employed to acquire images, and the area of active eWAT adipocytes was analyzed using the NDP.View 2 software (version 2.9.22). The area size of adipocytes in each experimental group was compared, and each image was further analyzed with the Image-Pro Plus software (version 6.0.0.260).

### 2.4. RNA-Seq Analysis and Biological Analysis of eWAT

Total RNA isolated from the eWAT underwent lysis via the TriZol method. The extracted RNA, stored in RNase-free water, served as the template for first-strand cDNA synthesis. This cDNA was subsequently amplified by full-length long-distance PCR (LD-PCR). The resulting double-stranded cDNA (ds cDNA) was purified using AMPure XP beads and quantified with a Qubit fluorometer. Following fragmentation by Covarissonication, the ds cDNA underwent end-repair, A-tailing, and adapter ligation for sequencing. After another AMPure XP purification step, fragments around 200 bp were isolated and enriched by PCR to construct a cDNA library. The library was quantified with Qubit 2.0 and diluted to 2 ng/µL. The Agilent 2100 Bioanalyzer assessed the library insert size, ensuring it matched expectations. Quantitative PCR (qPCR) then accurately determined the effective concentration, exceeding 2 nM and confirming library quality. Libraries meeting the required concentration and targeted data volume were sequenced on the HiSeq platform. Sequenced reads were aligned to the reference mouse genome (mm10). Gene expression levels were normalized using the fragments per kilobase of transcript per million mapped reads (FPKM) method. Gene Ontology (GO; http://www.geneontology.org accessed on 20 July 2024) was used for functional annotation, while the Kyoto Encyclopedia of Genes and Genomes (KEGG; http://www.genome.jp/kegg/ accessed on 20 July 2024) identified the associated pathways. Majorbio (Shanghai, China) assisted with sequencing and data annotation. The differential gene expression analysis utilized read count data for normalization, DESeq model-based *p*-value calculation, and false discovery rate (FDR) determination through multiple hypothesis testing. The DESeq software version 3.20 identified differentially expressed genes under various conditions. A less stringent criterion of twofold change and a *p*-value threshold of 0.05 was employed to filter these genes for biological analysis, focusing on functions with biological significance.

### 2.5. Lipid Extraction and Lipidomics Analysis of eWAT

eWAT was harvested from the mice. The tissues were rinsed with cold phosphate-buffered saline (PBS) to remove blood contamination and immediately snap-frozen in liquid nitrogen. The samples were stored at −80 °C until lipid extraction. eWAT samples were removed from −80 °C, and lipids were extracted using the MTBE method. Briefly, the tissues were homogenized in a cold mixture of MTBE, methanol, and water (5:1.5:1 *v*/*v*). The homogenate was shaken for 1 h at room temperature to induce phase separation. The organic phase was isolated by centrifugation at 10,000× *g* for 10 min at 4 °C. The organic phase was collected, the solvent was evaporated under a nitrogen stream, and the lipid extract was stored at −80 °C until analysis. The extracted lipids were subjected to an LC-MS/MS analysis. Chromatographic separation was performed using a C18 column with a mobile phase consisting of water containing 0.1% formic acid (mobile phase A) and acetonitrile containing 0.1% formic acid (mobile phase B). The lipid extracts were injected into an LC-MS/MS system, and lipid species were separated by gradient elution using electrospray ionization (ESI) in both the positive and negative ion modes. MS and MS/MS data were acquired, and the LipidSearch (Thermo Fisher, Waltham, MA, USA) software version 4.1 was employed for peak extraction, alignment, identification, and other analyses. A data matrix containing retention time, peak area, mass–charge ratio, and identification information was generated. To identify differential lipid metabolites, *t*-tests and variable importance in projection (VIP) scores from orthogonal partial least squares discriminant analysis (OPLS-DA) were utilized. The biological significance of differential lipids was further explored through personalized analyses, such as association analysis and cluster analysis.

### 2.6. Quantitative Real-Time PCR Analysis

eWAT was extracted from the mice, washed thrice with PBS, and stored at −80 °C until RNA extraction. Total RNA was isolated using TRIzol (Invitrogen, Carlsbad, CA, USA), quantified spectrophotometrically at 260 nm, and stored at −80 °C. The mRNA expression levels of the target genes were assessed using quantitative real-time PCR (qRT-PCR) on a LightCycler (Vazyme Biotech, Nanjing, China) with the OneStep SYBR PrimeScript RT-PCRKit (Takara Bio, Tokyo, Japan). Following a melting curve analysis, the relative expression of the target genes was determined by the second derivative method and normalized to β-actin expression. The primer pairs for the target mRNAs are listed in Table 1.

### 2.7. Immunofluorescence Staining

Paraffin-embedded eWAT sections were deparaffinized, rehydrated, and subjected to antigen retrieval using sodium citrate buffer. Endogenous peroxidase activity was quenched with 3% hydrogen peroxide solution, followed by blocking with 5% bovine serum albumin (BSA) for 30 min at 37 °C. The sections were incubated with primary antibodies overnight at 4 °C, washed, and subsequently incubated with the appropriate fluorescently labeled secondary antibody (926-32211, Servicebio, Wuhan, China) for 30 min at 37 °C in the dark. The slides were sealed with an anti-quench fluorescence cartridge containing DAPI (4′,6-diamidino-2-phenylindole), and images were acquired using a DS-Fil digital camera mounted on a Nikon Eclipse 80i fluorescence microscope (Nikon, Tokyo, Japan). The following primary antibodies were employed: AANAT (ab3505, Abcam, Waltham, MA, USA), MTNR1A (PA5-75749, Thermofisher, Beijing, China), CEBPB (AF6202, Affinity Biosciences, Changzhou, China), MMP9 (AF5228, Affinity Biosciences, Changzhou, China), IL-1β (16806-1-AP, Proteintech, Wuhan, China), and NF-κB (8242, Cell Signaling Technology, Danvers, MA, USA).

### 2.8. Western Blot Analysis

Total protein was extracted from the mouse eWAT tissue using a total protein extraction kit (KGB5303, KeyGEN, Nanjing, China), and protein concentration was determined using a BCA protein assay kit (BL521A, Biosharp, Hefei, China). Proteins were separated by SDS-PAGE gel electrophoresis and transferred to PVDF membranes (1620177, Bio-Rad, Hercules, CA, USA). The membranes were blocked using 3% BSA and incubated with primary antibodies overnight at 4 °C, and after TBST washing, the membranes were incubated with secondary antibodies for 1.5 h at room temperature. Finally, images were scanned and analyzed using the Odyssey Infrared Imaging system (Li-COR Biosciences, Lincoln, NE, USA). The primary antibodies used were NLRP3 (BF8029, Affinity Biosciences, 1:1000), IL-1β (16806-1-AP, Proteintech, 1:1000), CEBPB (AF6202, Affinity Biosciences, 1:1000), PINK1 (23274-1-AP, Proteintech, 1:1000), PARKIN (14060-1-AP, Proteintech, 1:1000), MMP9 (AF5228, Affinity Biosciences, 1:1000), NF-κB (8242, Cell Signaling Technology, 1:1000), phospho-NF-κB (3033, Cell Signaling Technology, 1:1000), MTNR1A (PA5-75749, Thermofisher, 1:1000), and AANAT (ab3505, ABCAM, 1:1000).

### 2.9. Measurement of Glutathione

After standing for 1 h, the freshly collected mouse blood was centrifuged at 3000 rpm for 10 min at 4 °C, and the upper serum was carefully collected and stored at −80 °C. Serum GSH levels were assessed using a reduced glutathione (GSH) assay kit (A006-1-1, Nanjing Jiancheng Bioengineering Institute, Nanjing, China). Adipose tissue was processed with a ratio of 1 g of tissue to 9 mL of normal saline, and then mechanically mixed in an ice-water bath. The supernatant was collected and centrifuged again at 4 °C and 3000 rpm for 10 min. Finally, the GSH level in the adipose tissue was measured following the instructions provided with the GSH assay kit.

### 2.10. ELISA for Measurement of Cytokines

The concentrations of TNF-α and IL-1β in the mouse serum were measured by the commercial ELISA kits according to the manufacturer’s instruction (RK00006, RK00027, ABclonal, Wuhan, China). The absorbance of each well was detected at 450 nm with a microplate reader. Each average value represents the values of triplicate experiments.

### 2.11. Measurement of Melatonin

Serum and supernatant samples were collected for melatonin measurement by HPLC- mass spectrometry, as previously described by He et al. [34]. Quantitative ion chromatography–mass spectrometry analysis of melatonin standard solutions is presented in Appendix C.

### 2.12. Transmission Electron Microscopy

Fresh eWAT samples were diced into 1 mm^3^ cubes and fixed in a 2.5% glutaraldehyde solution at 4 °C. Following post-fixation with osmium tetroxide and phosphoric acid, the samples were dehydrated in a graded ethanol series, followed by acetone, and embedded in epoxy resin. Ultrathin sections (60 nm) were cut, stained with 2% uranyl acetate and lead citrate solutions, and examined using a transmission electron microscope (Hitachi HT7800, Hitachi, Japan).

### 2.13. Statistical Analysis

Statistical analyses were performed using GraphPad Prism 10 (GraphPad Software, San Diego, CA, USA). Unless otherwise specified, the data are expressed as mean ± standard error of the mean (SEM). An analysis of variance (ANOVA) was used to assess normality among the groups, followed by Dunnett’s test for multiple comparisons. All statistical tests were performed using the SPSS Statistics 26.0 software. A *p*-value < 0.05 was considered statistically significant.

## 3. Results

### 3.1. Aging and High-Fat Diet Lead to Increased Lipid Deposition and Inflammation in eWAT

**Figure 1:**
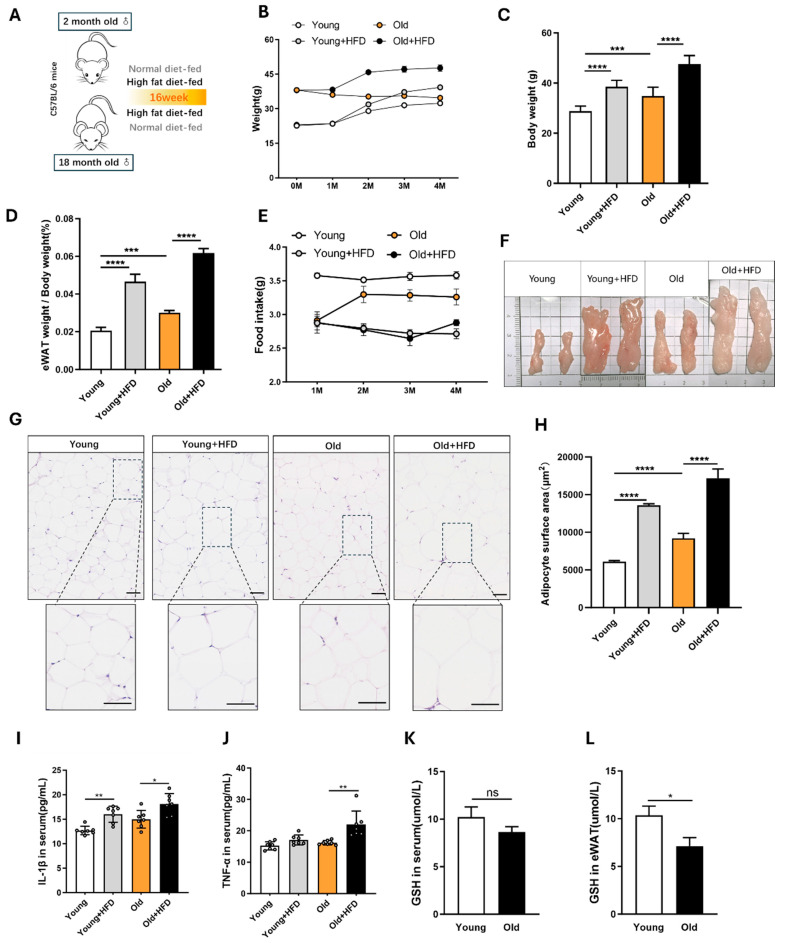
Effect of high-fat diet (HFD) and aging on eWAT of mice. (**A**) Schematic diagram of the experimental procedure; (**B**) body weight curves; (**C**) average weights of the mice; (**D**) average eWAT weight/weight of the mice; (**E**) mean food consumption; (**F**) representative images of the eWAT of the mice; (**G**) histopathological assessment of the eWAT using H&E staining; (**H**) adipocyte surface area; (**I,J**) IL-1β and TNF-α level in serum of mice detected by ELISA; (**K**) GSH level in serum; (**L**) GSH in eWAT; (**L**) data are expressed as the mean ± SEM (n = 7). All the data are represented as mean ± SEM. * *p* < 0.05, ** *p* < 0.01, *** *p* < 0.001, and **** *p* < 0.0001.

**Figure A1:**
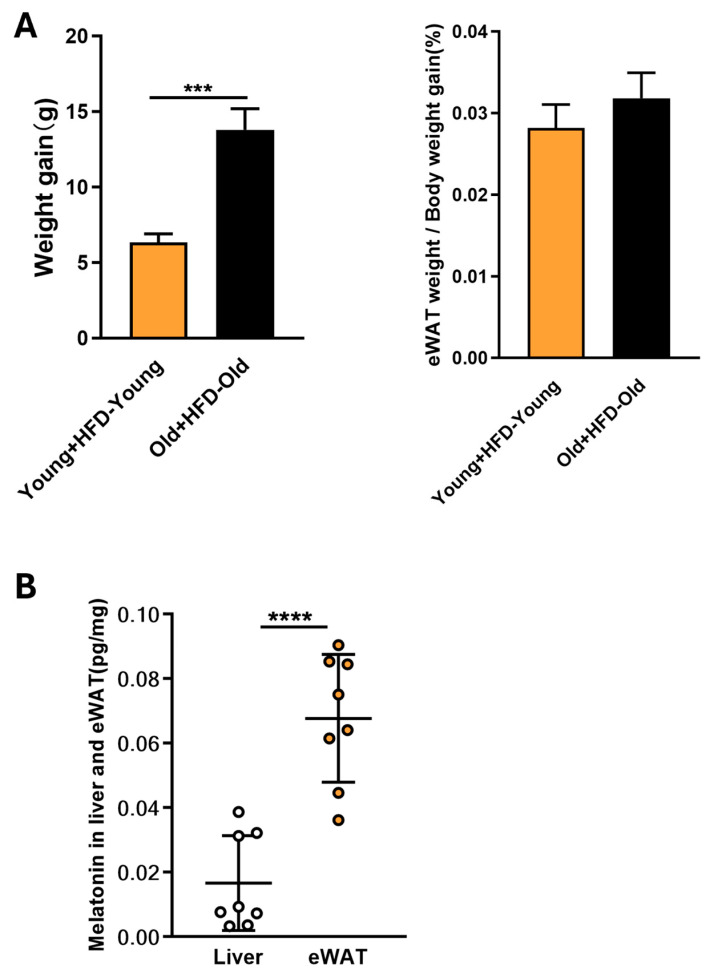
High-fat-induced increases in body weight and eWAT weight were exacerbated in aged mice. (**A**) Average weight gain (left) and eWAT weight/weight gain (right) of mice; (**B**) melatonin levels in liver and eWAT detected by liquid chromatography. All data are represented as mean ± SEM. *** *p* < 0.001, **** *p* < 0.0001.

To evaluate the effects of aging and a high-fat diet on eWAT, we divided the young (2-month-old) and aged (18-month-old) mice into two groups each: one group was fed a high-fat diet and the other a normal diet (ND) for 16 weeks (Figure 1A). Both the young HFD and aged HFD mice exhibited significant increases in body weight and eWAT index (Figure 1C,D). Notably, the increases in body weight and eWAT index were greater in the aged HFD group compared to the young HFD group (Figure A1A). Food intake did not differ significantly between the young and aged mice on the HFD but was reduced in the aged mice compared to the young mice on the ND (Figure 1E). The histological analysis of eWAT revealed that the combination of aging and HFD resulted in significantly enlarged fat tissue with increased area per unit (Figure 1G,H). Additionally, aged mice on the HFD showed increased inflammation as evidenced by the elevated serum levels of IL-1β and TNF-α (Figure 1I,J). While the IL-1β levels were significantly higher in the young HFD group compared to the young controls, the TNF-α levels remained unchanged. These findings confirmed more severe eWAT hypertrophy and inflammation in the aged mice. We further measured the GSH levels in the serum and eWAT of the young and aged mice. Although the serum GSH levels did not decrease significantly in the aged mice, the GSH levels in the eWAT were significantly reduced (Figure 1K). These results indicate that both aging and a high-fat diet exacerbate obesity and eWAT hypertrophy in mice, with a more pronounced negative effect of HFD in aged mice.

### 3.2. Lipidomics Reveals High-Fat Diet-Induced Lipid Deposition in eWAT of Aged Mice

**Figure 2:**
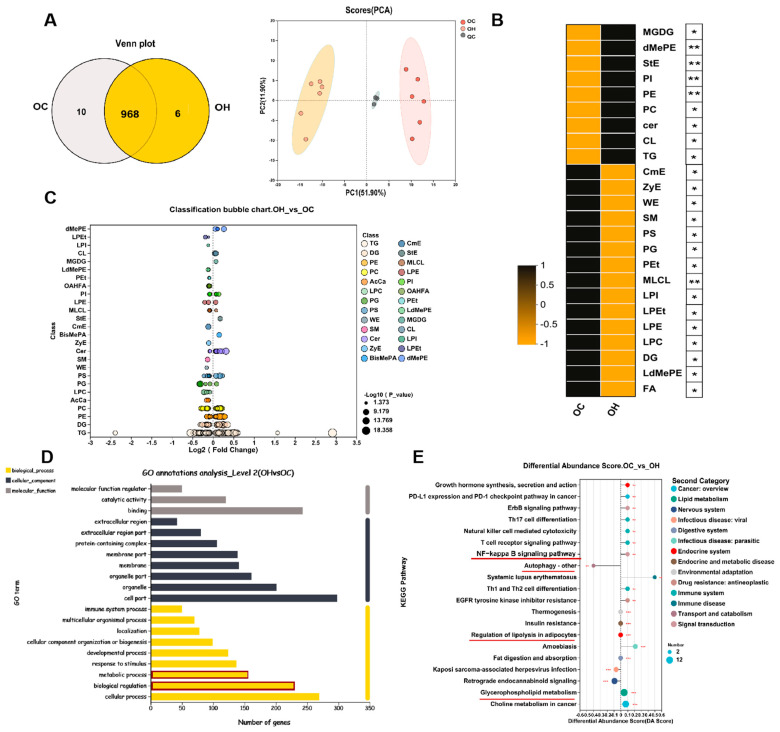
Changes in the overall lipid composition and distribution in the eWAT of the OC and OH group. (**A**) Left: Venn diagram of shared differentially expressed Lipidomics analysis. Right: the numbers of lipid classes and scores (PCA) in the OC and OH groups. (**B**,**C**) Heatmap for Lipid in OC and OH groups classification bubble chart; (**D**) Lipid ontology (GO) annotations analysis in OC and OH groups; (**E**) KEGG enrichment analysis in the OC and OH groups. Data are expressed as the mean ± SEM (n = 6). All the data are represented as mean ± SEM. * *p* < 0.05, ** *p* < 0.01, and *** *p* < 0.001.

To investigate lipid alterations in the eWAT of the aged mice following a high-fat diet, we conducted a lipidomics study on the epididymal fat of both the aged and aged HFD mice. We observed a significant upregulation of several lipid species in the aged HFD fat tissue, including sterol esters (StEs), phosphatidylinositol (PI), phosphatidylethanolamine (PE), phosphatidylcholine (PC), ceramide (Cer), cardiolipin (CL), and triacylglycerol (TG). Conversely, cholesteryl ester (ChE), zymosterol ester (ZyE), wax ester (WE), sphingomyelin (SM), phosphatidylserine (PS), phosphatidylglycerol (PG), phosphatidylethanol (Pet), monolysocardiolipin (MLCL), and lysocardiolipin (LPI) were significantly downregulated (Figure 2B,C). Elevated levels of StEs, PI, PE, TG, and PC are known to promote excessive lipid accumulation in adipose tissue, while elevated Cer is associated with inflammation in fat tissue. SM is involved in anti-inflammatory responses, and PG and MLCL, predominantly found in mitochondrial membranes, are crucial for mitochondrial health. The Gene Ontology (GO) analysis revealed that differentially expressed lipids were predominantly enriched in the pathways related to cell membrane signaling, immune processes, and metabolic regulation (Figure 2D). The KEGG pathway analysis indicated that the majority of the enriched pathways were associated with NF-κB signaling, autophagy, lipid metabolism, and glycerophospholipid metabolism. These results suggest that a high-fat diet significantly alters lipid metabolism in aged mice, promoting lipid accumulation and inflammation, which may be associated with reduced mitochondrial health.

### 3.3. Transcriptomics Reveals High-Fat Diet Alters Gene Regulation in eWAT of Aged Mice

**Figure 3:**
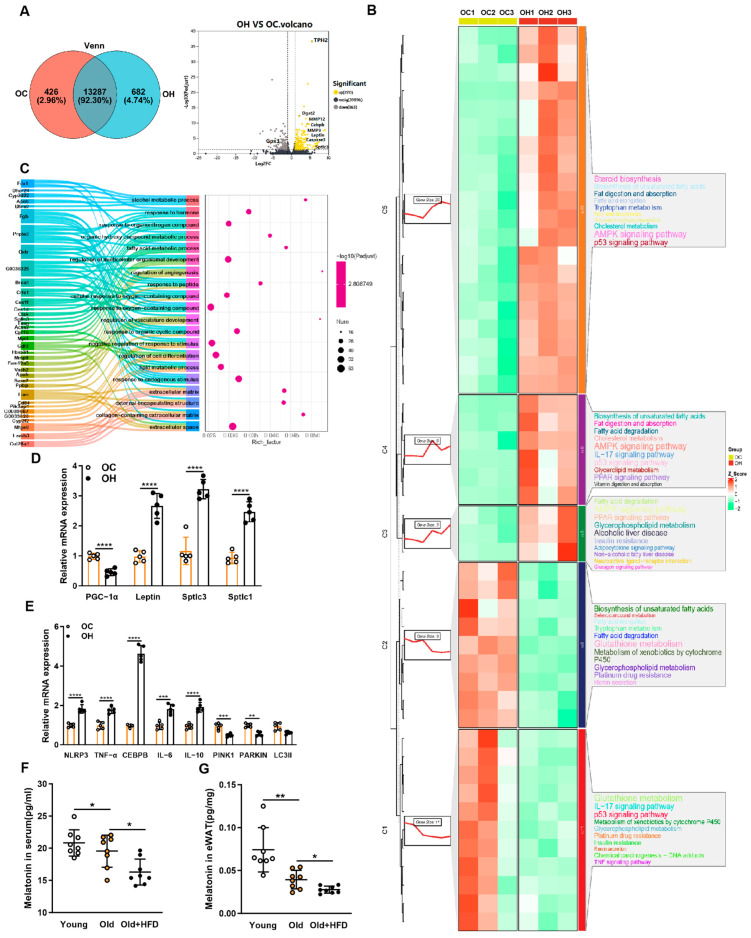
Characterization of mice eWAT transcriptomic characteristics. (**A**) Left: Venn diagram of shared differentially expressed genes. Right: volcano plot of differentially expressed genes in the OC and OH groups. The yellow dots represent upregulated genes, black dots represent downregulated genes, and gray dots represent genes with no significant differences. * *p* < 0.05, ** *p* < 0.01, *** *p* < 0.001, and **** *p* < 0.0001. (**B**) Heatmap and hierarchical clustering of genes involved in different stages. The colors represent the relative gene expression values after normalization adjustments. The red and green colors refer to up- and downregulation, respectively. (**C**) Strategy for the enrichment analysis of metabolites in the highlighted cluster with MetaboAnalyst 5.0 based on the KEGG and SMPDB (the Small Molecule Pathway Database) database. (**D**,**E**) PGC-1α, Leptin, Sptlc3, Sptlc1, NLRP3, TNF-α, CEBPB, IL-6, IL-10, PINK1, Parkin, and LC3II mRNA transcription assays, mouse eWAT from the OC group (normal diet aged mice) and OH group (HFD aged mice) followed by qPCR analysis (n = 6, mean ± SEM). (**F**) Serum melatonin levels in the groups Young, Old, and Old + HFD. (**G**) eWAT melatonin levels in the groups Young, Old, and Old + HFD. All the data are represented as mean ± SEM. * *p* < 0.05, ** *p* < 0.01, and **** *p* < 0.0001.

**Figure A2:**
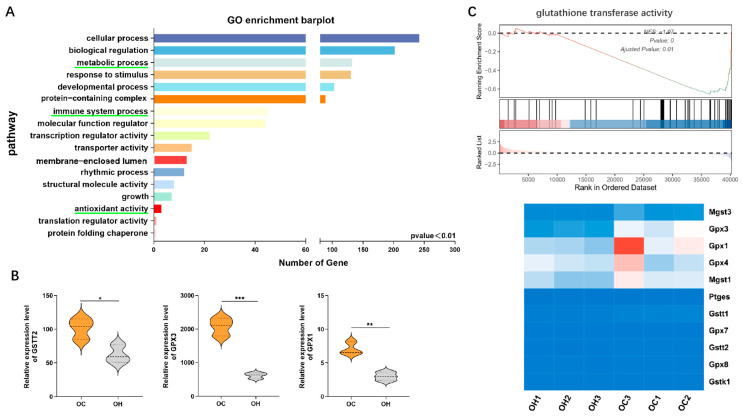
Changes in antioxidant-related genes in eWAT of the OC and OH groups. (**A**) Transcriptome GO enrichment barplot of annotations analysis in OC and OH groups. (**B**) Violin plot shows the relative expression levels of three antioxidant-related genes (GPST2, GPX3, and GPX4) in the OC and OH groups in the transcriptome. (**C**) Top left: GSEA of genes regulating glutathione transferase activity, enrichment score NES = −1.97, *p* < 0.01; bottom right: ranked gene list of GSEA. All the data are represented as mean ± SEM. * *p* < 0.05, ** *p* < 0.01, and *** *p* < 0.001.

To identify the direct targets regulated by a high-fat diet in the eWAT of the aged mice, we performed RNA sequencing on the eWAT tissues from the aged mice treated with either a high-fat diet or a normal diet. We identified 383 differentially expressed genes with a fold change greater than two (Figure 3A). The most significantly altered gene was TPH2 (tryptophan hydroxylase 2), an enzyme involved in the synthesis of serotonin (5-HT), a precursor of melatonin. Other significantly upregulated genes included Dgat2, MMP12, MMP9, CEBPB, leptin, caspase3, and sptlc3, while glutathione peroxidase GPX3 was significantly downregulated (Figure 3A). These genes are associated with lipid metabolism and inflammation. The differentially expressed genes were clustered into five functional categories using K-means: Steroid Biosynthesis, Fat Digestion and Absorption, Biosynthesis of Unsaturated Fatty Acids, Tryptophan Metabolism, and others including glutathione metabolism and TNF signaling pathway (Figure 3B). A Gene Set Enrichment Analysis (GSEA) was used to investigate changes in glutathione metabolism. The results showed a downregulation of genes that regulate glutathione transferase activity in the aged mice fed a high-fat diet (Figure A2C). The analysis of the top 20 enriched terms revealed significant involvement in multicellular organismal development, response to stimulus, cell differentiation, and lipid metabolic processes. RT-PCR further confirmed that the high-fat diet significantly upregulated inflammation-related genes (NLRP3, TNF-α, CEBPB, IL-6, and IL-10) and downregulated genes related to mitochondrial autophagy (PINK1 and PARKIN) (Figure 3E). The GO functional enrichment analysis revealed significant enrichment in the pathways related to immune system processes and antioxidant activity (Figure A2A). These results indicate that a high-fat diet modifies gene expression in the eWAT of aged mice, activating inflammatory pathways and impairing antioxidant defense mechanisms.

### 3.4. Correlation Analysis of Lipidomics and Transcriptomics Reveals Effects on Metabolic Regulation in the eWAT of Aged Mice Fed a High-Fat Diet

**Figure 4:**
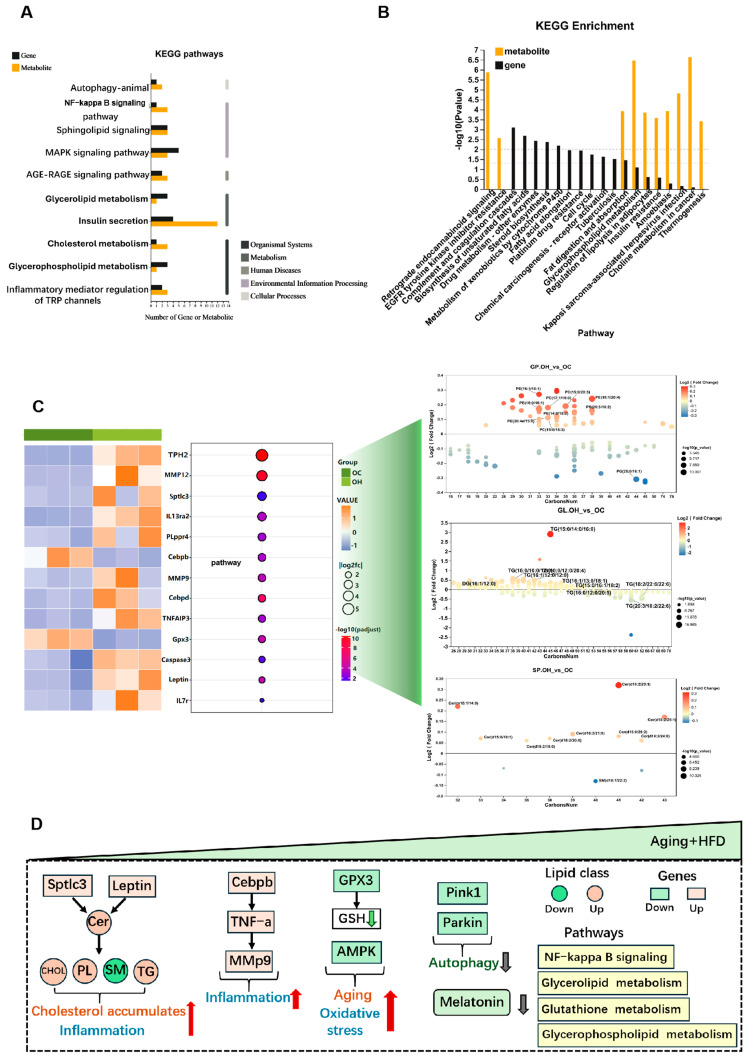
Correlation analysis of transcriptome and lipidomics revealed the changes in the regulation of eWAT by a high-fat diet in the aged mice. (**A**,**B**) Correlation KEGG enrichment analysis for differential genes and lipid molecules. (**C**) From left to right: heatmap of the genes with the most significant differences between the OH and OC group; the molecular changes in different kinds of lipids between the OH and OC group; and correlation analysis of genes and lipids. (**D**) Summary of the regulatory pathway changes in eWAT through lipidome and transcriptome.The red arrow in the figure indicates upregulation of expression and the green arrow indicates downregulation.

Next, we performed a combined lipidomics and transcriptomics analysis of eWAT to investigate the regulatory pathways affected by both genes and lipid molecules under HFD conditions in the aged mice. The KEGG enrichment analysis revealed that the differentially expressed genes and metabolites were predominantly enriched in the pathways related to autophagy, NF-κB signaling, phospholipid metabolism, and glycerophospholipid metabolism (Figure 4A,B). Correlating the differential gene expression with the lipidomics data, we found a significant upregulation of SPTLC3. As a key enzyme in ceramide (Cer) synthesis, the upregulation of SPTLC3 leads to increased Cer levels. Elevated leptin levels were associated with increased triglycerides (TGs), consistent with our lipidomics results (Figure 4C). Among the transcriptomic findings, TPH2 emerged as the most significantly upregulated gene. TPH2, an enzyme responsible for converting tryptophan into 5-hydroxytryptophan (5-HTP), a precursor for melatonin, prompted us to examine melatonin levels. The liquid chromatography analysis revealed significantly reduced melatonin levels in both the serum and adipose tissue of the aged mice, with further reductions in the HFD-fed aged mice (Figure 3F,G). No significant differences in the liver melatonin levels were found between the young and aged mice (Figure A1B). Given that melatonin can promote GPX expression and raise GSH levels, as well as exert anti-inflammatory and antioxidant effects, these findings suggest that under the combined effects of aging and HFD, the upregulation of genes such as SPTLC3 and leptin promotes Cer accumulation, thereby increasing cholesterol and TG levels. Concurrently, the upregulation of CEBPB, TNF-α, and MMP9, along with the downregulation of GPX and reduced GSH levels, exacerbates inflammation, oxidative stress, and aging. Additionally, the reduced levels of PINK1 and PARKIN indicate diminished mitochondrial autophagy, while decreased melatonin levels may contribute to the increased fat deposition in the aged HFD mice (Figure 4D).

### 3.5. Melatonin Rescues Lipid Accumulation in the eWAT of Aged Mice Fed Ahigh-Fat Diet

**Figure 5:**
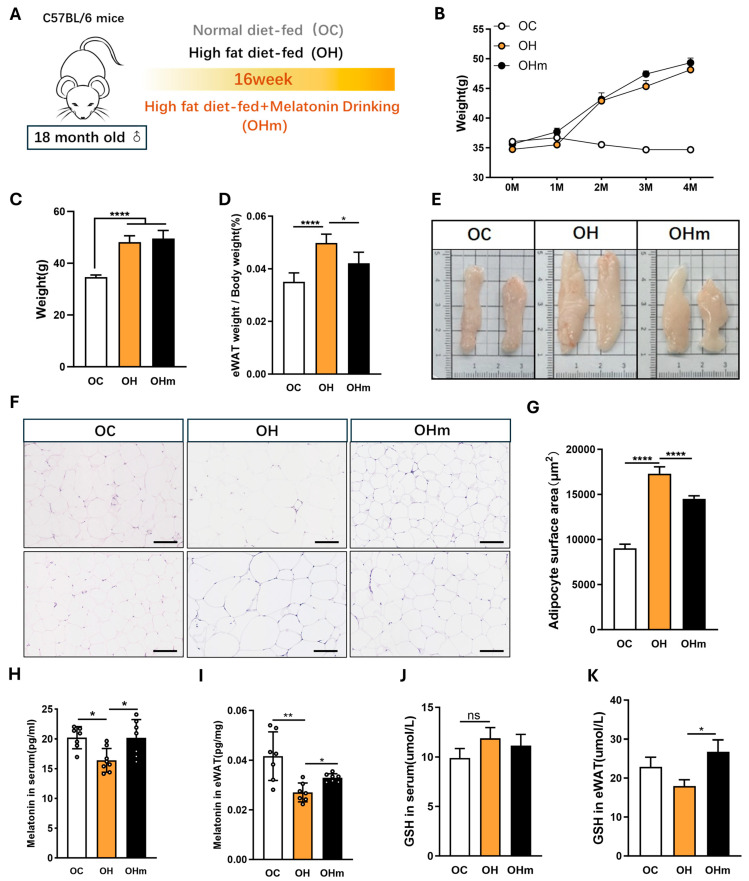
Effect of melatonin on the eWAT of the aged mice with high-fat diet. (**A**) Schematic diagram of the experimental procedure; (**B**) body weight curves; (**C**) average weights of the mice; (**D**) average eWAT weight/weight of the mice; (**E**) representative images of the eWAT of the mice; (**F**) histopathological assessment of the eWAT using H&E staining; (**G**) adipocyte surface area; (**H**,**I**) melatonin level in serum and eWAT detected by liquid chromatography; (**J**) GSH level in serum; (**K**) GSH in eWAT. All the data are represented as mean ± SEM. ^ns^ *p* > 0.05, * *p* < 0.05, ** *p* < 0.01, and **** *p* < 0.0001.

To determine whether melatonin can rescue lipid accumulation and inflammation in the eWAT of the aged mice subjected to a high-fat diet (HFD), we divided the aged mice into three groups: OC, OH, and OHm over a period of 16 weeks (Figure 5A). Body weight was not significantly different between the OHm and OH groups, but the fat index was significantly reduced in the OHm group (Figure 5B,C). The HE staining of eWAT showed a significant reduction in fat area per unit in the OHm mice compared to the OH mice (Figure 5D). The liquid chromatographic analysis showed that melatonin levels were significantly higher in the serum and eWAT of the OHm mice compared to the OH mice (Figure 5H,I), confirming that oral melatonin increases the levels of melatonin in fat and serum. In addition, the ELISA results showed that melatonin can rescue the levels of GSH in the fat of the aged mice on a high-fat diet (Figure 5J), although no significant changes were observed in the serum (Figure 5K). These results suggest that melatonin supplementation can ameliorate lipid accumulation and enhance GSH levels in the eWAT of aged mice on a high-fat diet.

### 3.6. Melatonin Increases the Expression of AANAT and MTNR1A in the eWAT of Aged Mice Fed a High-Fat Diet

**Figure 6:**
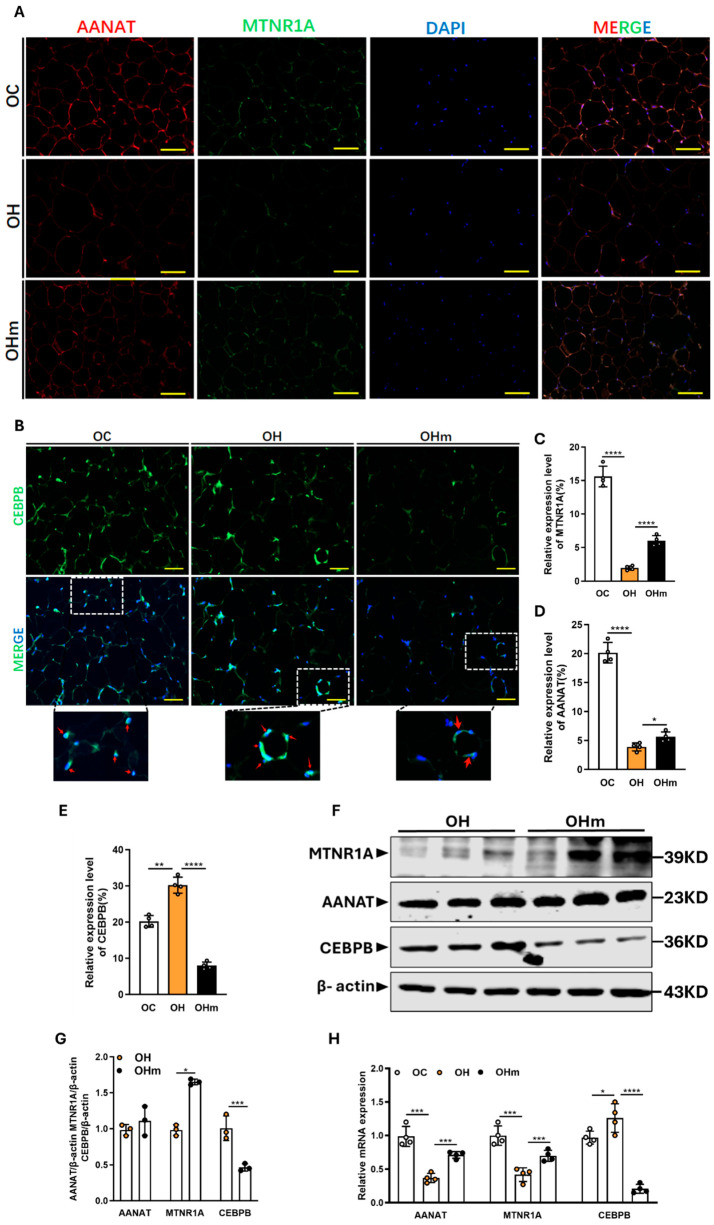
Melatonin increases AANAT and MTNR1A expression in eWAT. (**A**) AANAT and MTNR1A in eWAT detected by immunofluorescence double staining (scale bar is 50 μm); (**B**) immunofluorescence analysis of CEBPB in eWAT (scale: 50 μm); (**C**–**E**) statistical graph of MTNR1A,AANAT and CEBPB positive expression rate; (**F**) Western blot analysis of MTNR1A protein expression in eWAT; (**G**) statistical graph of protein expression; (**H**) relative expression of MTNR1A, AANAT and CEBPB mRNA expression by qPCR analysis. All data are represented as mean ± SEM. * *p* < 0.05, ** *p* < 0.01, *** *p* < 0.001, and **** *p* < 0.0001.

We then sought to elucidate the mechanism by which melatonin rescues eWAT damage in the aged mice on an HFD by examining the expression of key melatonin-related proteins. The immunofluorescence results showed a significant increase in the expression of the melatonin synthesis enzyme AANAT and the membrane receptor MTNR1A in the eWAT of the OHm mice compared to the OH mice (Figure 6A,C,D). The Western blot (WB) analysis further confirmed that the protein levels of MTNR1A and AANAT were significantly higher in the OHm group (Figure 6F,G). In addition, the mRNA expression levels of MTNR1A and AANAT in the eWAT of the OHm mice were also significantly increased compared to the OH mice. These results suggest that melatonin rescues eWAT in aged mice under HFD through its receptor MTNR1A. Furthermore, the decreased expression of melatonin synthesis enzymes and lower melatonin levels in eWAT under HFD suggest that a high-fat diet suppresses the synthesis of melatonin in the eWAT.

### 3.7. Melatonin Improves Mitochondrial Autophagy and Inflammation in eWAT of Aged Mice on a High-Fat Diet

**Figure 7:**
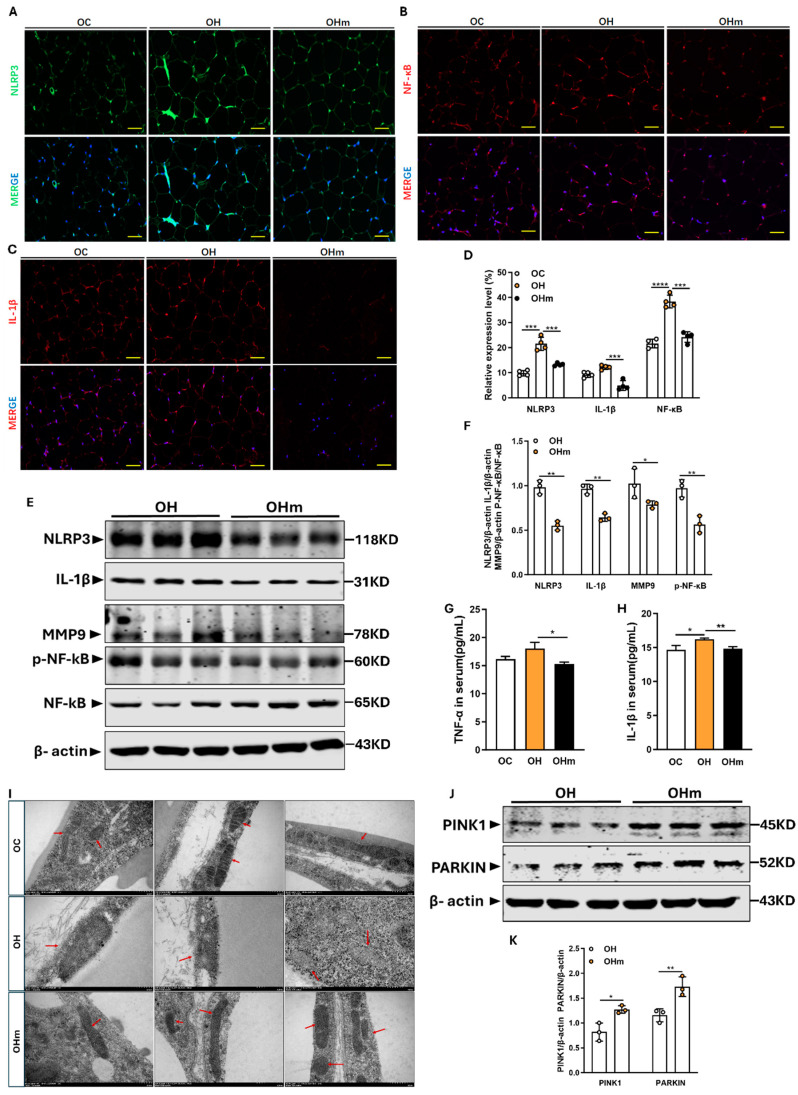
Melatonin improves mitochondrial autophagy and inflammation in eWAT. (**A**–**C**) immunofluorescence analysis of NLRP3, NF-κB, and IL-1β in eWAT (scale: 50 μm); (**D**,**F**) statistical graph of NLRP3, NF-κB, and IL-1β positive expression rate; (**E**) Western blot analysis of NLRP3, NF-κB, MMP9, and IL-1β protein expression in eWAT; (**G**,**H**) IL-1β, TNF-α level in serum of mice detected by ELISA; (**I**) transmission electron microscope picture of eWAT (scale: 50 nm); (**J**) Western blot analysis of PINK1 and PARKIN protein expression in eWAT; (**K**) statistical graph of protein expression. The red arrow points to the mitochondria. All data are represented as mean ± SEM. * *p* < 0.05, ** *p* < 0.01, *** *p* < 0.001, and **** *p* < 0.0001.

To confirm the mechanism by which melatonin protects eWAT, we first assessed its effect on inflammatory markers. The ELISA results showed that supplementation with melatonin significantly reduced the serum levels of TNF-α and IL-1β in the aged mice on an HFD compared to those on an HFD without melatonin (Figure 7G,H). To further investigate the effect of melatonin on inflammation in eWAT, the immunofluorescence and Western blot (WB) analyses revealed a significant reduction in the protein expression of NLRP3, NF-κB, and IL-1β in the eWAT of the melatonin-supplemented mice compared to those on HFD alone (Figure 7A–C,E). Additionally, the WB results showed a significant downregulation of the inflammatory marker MMP9 (Figure 7E,F). CEBPB, a regulator of inflammatory factors and TNF-α, was significantly upregulated in the eWAT of the HFD mice, as confirmed by transcriptomic data (Figure 3A). The immunofluorescence and WB results indicated that melatonin effectively downregulated CEBPB protein levels (Figure 6B,F). Mitochondria are crucial for oxidative stress resistance and metabolic regulation in adipocytes, and melatonin has been reported to protect mitochondrial function. The transmission electron microscopy of eWAT revealed that HFD-induced changes in mitochondrial morphology, such as outer membrane damage and the loss of cristae, were attenuated by melatonin, which preserved normal mitochondrial structure and outer membrane integrity (Figure 7I). The accumulation of damaged mitochondria may be related to impaired mitochondrial autophagy, a notion supported by the WB results showing a significant increase in the mitochondrial autophagy markers PINK1 and PARKIN following melatonin supplementation (Figure 7J). These results demonstrate that melatonin enhances mitochondrial autophagy and preserves mitochondrial function. In addition, melatonin attenuates inflammation in the eWAT of aged mice on HFD by downregulating CEBPB and genes downstream of the inflammatory response, thereby ameliorating the overall inflammatory state.

## 4. Discussion

Aging and a high-fat diet have independent effects on WAT function. However, their combined effects are poorly understood. Aging primarily affects fatty acid saturation metabolism in eWAT. It increases fatty acid saturation and decreases specific lipid types, such as triglycerides and phospholipids. Aging may also reduce the hydroxy fatty acids in epididymal fat, altering cholesterol and triglyceride metabolism, and affecting signaling pathways such as mTOR and AMPK [36,37]. Conversely, a high-fat diet increases eWAT lipid content, particularly triglycerides and cholesteryl esters; alters fatty acid composition [38]; and modifies lipid metabolism-related gene expression, including the upregulation of PPARγ and SREBP-1c [39]. It also affects mTOR and AMPK pathways and exacerbates inflammation through NF-κB-related pathways [40,41,42]. Our experiments with young and old mice on high-fat diets confirmed that both aging and a high-fat diet lead to obesity, increased adipose tissue, and elevated inflammatory markers. The combined effects of aging and a high-fat diet exacerbate these changes. The lipidomics analysis revealed that in the aged mice on a high-fat diet, levels of StE, PI, PC, Cer, and TG were significantly elevated, while SM, PS, PG, and MLCL were decreased. Increased levels of StE, PI, PE, TG, and PC promote excessive lipid accumulation, while elevated Cer promotes inflammation [43]. SM may play a role in anti-inflammatory responses [44]. The transcriptomic analysis showed significant alterations in genes related to inflammation, NF-κB signaling, autophagy, and lipid metabolism. The integration of lipidomics and transcriptomics highlights that lipidomic changes correlate with notable shifts in gene expression associated with these processes.

Using transcriptomic analysis, we identified TPH2 as the most significantly upregulated gene. TPH2 is an enzyme responsible for the hydroxylation of tryptophan to produce 5-HTP, which is a substrate for melatonin synthesis [45]. Nevertheless, we observed that melatonin levels in both the serum and adipose tissue were significantly lower in the aged mice compared to the young mice. Furthermore, the decrease in melatonin levels in eWAT was more pronounced in the mice subjected to both a high-fat diet and aging. No significant difference in melatonin levels was found between the livers of the young and old mice (Figure A1B). These findings suggest that melatonin’s impact on eWAT is particularly crucial, and the reduced melatonin levels in the elderly mice on a high-fat diet may contribute to more severe metabolic dysregulation in eWAT. For animals, melatonin is also a hormone that regulates biological rhythms, sleep, inflammation, glucose metabolism, and cancer in addition to its antioxidant effects [46,47,48]. An inhibitory aspect is also evident. Based on these characteristics, the identification of the novel physiological functions of melatonin has been the focus of research in this field. The role of melatonin as a regulatory molecule in metabolic health is increasingly recognized [49], and it has been found that serum melatonin levels are significantly reduced in aged mice, which is consistent with our findings [50]. However, the changes in melatonin in the adipose tissue of aged mice have not been reported. At the same time, many studies have found that melatonin can reduce body weight and ameliorate obesity in mice on a high-fat diet [51]. In the regulation of adipose tissue, melatonin has been found to increase m^6^A RNA demethylation in adipocytes and inhibit resistin production, thereby improving steatosis [52]. It has been found that the expression of the receptor MTNR1A is increased in young mice after melatonin supplementation [53], which is also similar to the results of this study. However, the difference is that the experimental subjects in this study were aged mice. The transcriptome analysis revealed that numerous inflammatory response-related pathways were upregulated in the eWATof high-fat diet (HFD)-fed mice, including several interleukin (IL) and tumor necrosis factor (TNF) family genes. Notably, melatonin supplementation resulted in a reduction in the IL-1β and TNF-α levels in the eWAT of the HFD-fed mice. Consistent with previous research, inflammatory markers from the IL and TNF families were similarly elevated in the eWAT transcriptome of the young mice on HFD [54], while melatonin supplementation led to their downregulation [55]. These findings suggest that HFD-induced eWAT inflammation occurs independently of age, though IL-1β and TNF-α levels were more pronounced in the aged HFD-fed mice, indicating heightened sensitivity to caloric excess in older animals. Collectively, these data, along with prior studies, support that melatonin mitigates eWAT inflammation in both young and aged mice under HFD conditions.

We observed that the aged mice on a high-fat diet had significantly reduced levels of GSH and melatonin. This finding suggests that the combined effects of a high-fat diet and aging significantly impair the antioxidant defense mechanisms. Antioxidants play a crucial role in maintaining cellular homeostasis and protecting cells from damage caused by oxidative stress. GSH is the primary non-enzymatic antioxidant of the cell and reduces oxidative stress by neutralizing free radicals [56]. In our study, the aged mice on a high-fat diet showed significantly decreased GSH levels, and GO functional enrichment analysis revealed significant enrichment in pathways related to immune system processes and antioxidant activity. At the same time, the transcriptomic analysis revealed a significant reduction in the expression of GSH activity-related genes such as GPST2, GPX3, and GPX4 (Figure A2B), suggesting that impaired lipid metabolism and inflammatory states suppress the antioxidant system. This finding is consistent with previous studies where a high-fat diet significantly reduced GSH levels and increased oxidative stress [57,58]. In addition, these changes in gene expression could further exacerbate oxidative stress, leading to cellular damage and loss of function. Notably, melatonin supplementation not only significantly increased GSH levels but also demonstrated that melatonin may reduce oxidative stress by modulating the antioxidant system, thereby mitigating the metabolic abnormalities induced by a high-fat diet and aging. As a multifaceted molecule with potent antioxidant and anti-inflammatory properties, melatonin offers a promising therapeutic approach for intervention in obesity and age-related diseases.

Overall, our study shows that melatonin supplementation significantly counteracts the adverse effects of a high-fat diet and aging. At present, there is no relevant study on the determination of melatonin levels in eWAT of elderly mice with a high-fat diet. It not only prevents weight gain but also reduces the fat index and fat area, attenuates lipid accumulation and inflammation, improves mitochondrial function, and enhances antioxidant defenses, demonstrating its potential therapeutic benefits. The upregulation of arylamine N-acetyltransferase (AANAT) and the melatonin receptor MTNR1A following supplementation suggests that melatonin acts through the MTNR1A receptor pathway, which regulates circadian rhythms and metabolic processes, thereby explaining its broad benefits. The exacerbated inflammatory response in the eWAT of the aged mice on a high-fat diet is consistent with the notion that both systemic inflammation and localized adipose inflammation are hallmarks of metabolic disease [59]. The reduction in GSH levels in adipose tissue highlights increased oxidative stress [60], a condition commonly associated with both aging and high-fat consumption, leading to impaired cellular function and increased susceptibility to metabolic disorders [61,62]. Moreover, melatonin’s downregulation of inflammatory factors such as TNF-α, NLRP3, NF-κB, IL-1β, and CEBPB underscores its potent anti-inflammatory properties, which may be crucial for preventing or alleviating metabolic inflammation. Given that mitochondria are key sites for melatonin synthesis and that melatonin promotes mitophagy [63,64], this enhances the removal of damaged mitochondria, thereby maintaining cellular health [65]. Studies suggest that melatonin activates mitophagy through pathways such as PINK1/PARKIN. Our findings of increased mitophagy in eWAT following melatonin treatment support the importance of mitochondrial quality control in adipose tissue health and highlight the role of autophagy-related pathways in mediating the beneficial effects of melatonin.

In mammals, melatonin was initially believed to be primarily synthesized and secreted by the pineal gland [22]. However, subsequent research has revealed that melatonin is also produced and released in various other organs and cells, including the brain, heart, adrenal gland, retina, lens, cochlea, lung, gastrointestinal tract, liver, kidney, thyroid, pancreas, thymus, and spleen. It has been confirmed that melatonin synthesis occurs in viscera such as the carotid artery and reproductive tract, as well as in endothelial cells and skin [22,23]. Of the total amount of melatonin produced by mammals, it is thought that perhaps less than 5% is synthesized by the pineal gland. The second source of melatonin comes from multiple tissues throughout the body, possibly in the mitochondria of these cells. This accounts for most of the melatonin produced by mammals and is associated with metabolic regulation [66]. Consequently, it has become evident that melatonin plays a crucial role in regulating numerous tissues and organs. Extensive studies have been conducted on melatonin across different tissues, with variations observed in its concentration levels. For instance, the content of melatonin differs between serum, nose, and lung of mice [67]. Based on this finding, it can be hypothesized that fat metabolism may rely more heavily on melatonin. This study specifically focuses on investigating the protective effects of melatonin against obesity in aged mice within eWAT. Some studies suggest that melatonin synthesis is defective in the pineal glands of certain C57BL/6 mouse strains. However, other studies have shown that although the synthesis of melatonin in the pineal gland may be impaired in these strains, melatonin can still be synthesized in other tissues, particularly in the immune system [68]. In addition, it has been shown in recent years that melatonin can be synthesized in most tissues and organs in mice, with mitochondria being the primary site of synthesis outside the pineal gland [24,69]. Many of these studies have been conducted in C57BL/6 mice. For example, in studies using AANAT knockout mice, melatonin levels were found to be significantly reduced in the ovaries of C57BL/6 mice [36]. In addition, melatonin has been found to reduce inflammation in the adipose tissue of young mice after HFD [70]. A large body of extensive research has also shown that melatonin levels decrease significantly with age and that rhythmicity is lost in older mice and humans [71]. Specifically, studies have shown that serum melatonin levels are significantly lower in 12-month-old C57BL/6 mice compared to younger mice, and that the levels of key enzymes involved in melatonin biosynthesis, such as AANAT/ASMT, are also reduced in aging mice [48]. Given these findings, we believe that young mice may not be the best model for studying age-related changes. Older mice are more appropriate for this type of research. However, young mice may be suitable for studying the underlying mechanism by which melatonin ameliorates HFD. Apart from studying its antioxidant and anti-inflammatory abilities, melatonin’s role as a circadian rhythm molecule may also impact the regulation of aging rhythms in mice. Additionally, the rhythmic patterns of each tissue may vary, and further investigation into the rhythmic behavior of melatonin within each tissue is warranted.

Our study has several limitations. The strain of C57BL/6J mice used in the experiments has not been genetically characterized for its ability to produce melatonin at the pineal level. For this reason, it is possible that the aging process and the responses to both diet and melatonin may have occurred in a strain-specific manner. In addition, the specific regulatory mechanisms of a high-fat diet on eWAT in mice need to be further investigated. Widening the age range of the experimental mice would provide more reliable and generalizable results.

Importantly, these findings could inform new intervention strategies for the prevention and treatment of obesity and related metabolic diseases, thereby improving the health and quality of life of the elderly population.

## 5. Conclusions

**Figure 8:**
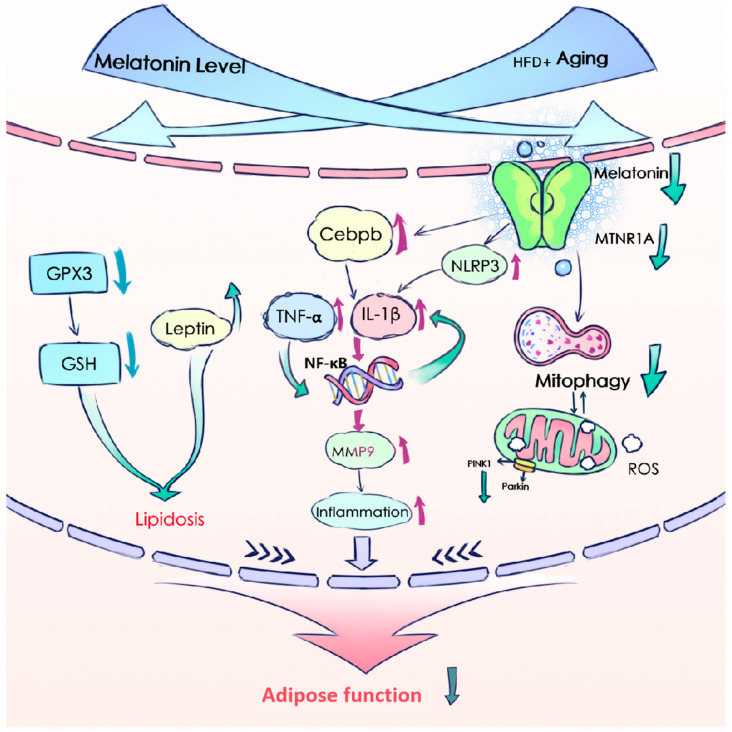
Summary of signaling pathways involved in adipocyte metabolic disorders caused by melatonin decline with aging and HFD in eWAT. Upward arrows indicate upregulated expression levels and downward arrows indicate downregulated expression levels.

In summary, our study provides a comprehensive analysis of how aging and a high-fat diet change lipid metabolism and inflammatory responses in eWAT. We found that reduced melatonin levels are associated with increased lipid deposition and inflammation, highlighting the potential of melatonin supplementation as a therapeutic strategy. Aging and HFD lead to decreased melatonin levels, the downregulation of the PINK1/PARKIN pathway, and reduced mitochondrial autophagy, which in turn promotes oxidative stress. In addition, melatonin modulates CEBPB to activate inflammatory pathways, thereby exacerbating inflammation (Figure 8). Our results suggest that melatonin supplementation may be an effective intervention by enhancing antioxidant defenses, regulating gene expression, and protecting mitochondrial function.

## Figures and Tables

**Table 1:** Primer pairs for mRNAs were as follows.

Genes	Sequence (5′-3′)
PGC1-α	F TGATGTGAATGACTTGGATACAGACA
	R GCTCATTGTTGTACTGGTTGGATATG
Leptin	F TCTCCGAGACCTCCTCCATCT
	R TTCCAGGACGCCATCCAG
NLRP3	F GCTAAGAAGGACCAGCCAGA
	R CAGCAAACCCATCCACTCTT
TNF-α	F CGTCAGCCGATTTGCTATCT
	R CGGACTCCGCAAAGTCTAAG
CEBPB	F TCGGGACTTGATGCAATCC
	R AAACATCAACAACCCCGC
SPTLC1	F TACGAGGCTCCAGCATACC
	R TCAGAACGCTCCTGCAACT
SPTLC3	F ACATCCATGAGTCCCGTAG
	R TCCATACCTCCAATGTTCC
IL-6	F AGTTGCCTTCTTGGGACTGA
	R TCCACGATTTCCCAGAGAAC
IL-10	F TGGACAACATACTGCTAACCGAC
	R CCTGGGGCATCACTTCTACC
AANAT	F CCACCAGTGCGTTTGAGA
	R AACCAGCCCAGTGACAGC
MTNR1A	F GCTGGTCATCCTGTCTGTGT
	R TATATTCCCTGAGTTCCTGAGCTTC
β-Actin	FGCACCGTCAAGGCTGAGAAC
	R TGGTGAAGACGCCAGTGGA

**Figure A3:**
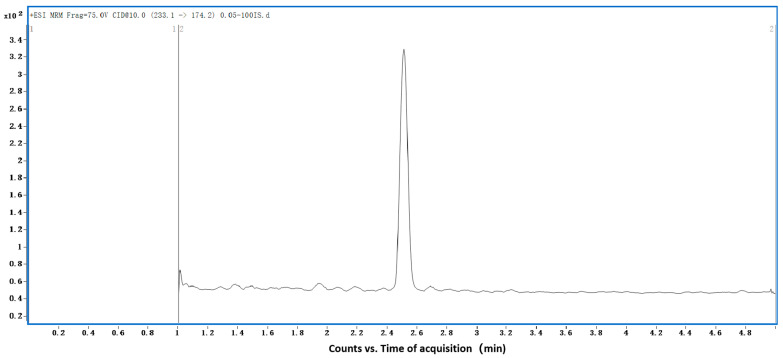
The melatonin content was detected by liquid chromatography, and the characteristic ion mass chromatograph of the melatonin standard solution was used (0.05 μg/L).

## Data Availability

Correspondence and requests for materials should be addressed to Zhaowei Cai.
